# 

**Published:** 1897-01

**Authors:** 


					﻿Southern Medical Record.
A Monthly Journal of Medicine and Surgery.
Vol. XXVII.
ATLANTA, GA., JANUARY, 1897,
No.I.
BERNARD WOLFF, M. D., Editor.
Associate Editors :
A. W. GRIGGS, M. D.
WILLIS F. WESTMORELAND, M. £>.
j. McFadden gaston, m. d.	\/ .
FERMS: $1.00 Per Annum; Single Copy, 10Cents.
Contents on Page 6.
PUBLISHED ON THE FIRST OF EACH MONTH.
Entered at the Post-Office at Atlanta, Ga., as Second-Class Matter.
The Foote & Davies Co.. Atlanta. Ga;
				

